# Obesity-Related Eating Behaviors Are Associated with Higher Food Energy Density and Higher Consumption of Sugary and Alcoholic Beverages: A Cross-Sectional Study

**DOI:** 10.1371/journal.pone.0077137

**Published:** 2013-10-18

**Authors:** Maritza Muñoz-Pareja, Pilar Guallar-Castillón, Arthur E. Mesas, Esther López-García, Fernando Rodríguez-Artalejo

**Affiliations:** 1 Department of Preventive Medicine and Public Health, Universidad Autónoma de Madrid/IdiPaz, CIBERESP, Madrid, Spain; 2 Departamento de Saúde Coletiva, Universidade Estadual de Londrina, Londrina, Brazil; Monash University, Australia

## Abstract

**Objectives:**

Obesity-related eating behaviors (OREB) are associated with higher energy intake. Total energy intake can be decomposed into the following constituents: food portion size, food energy density, the number of eating occasions, and the energy intake from energy-rich beverages. To our knowledge this is the first study to examine the association between the OREB and these energy components.

**Methods:**

Data were taken from a cross-sectional study conducted in 2008–2010 among 11,546 individuals representative of the Spanish population aged ≥18 years. Information was obtained on the following 8 self-reported OREB: not planning how much to eat before sitting down, eating precooked/canned food or snacks bought at vending machines or at fast-food restaurants, not choosing low-energy foods, not removing visible fat from meat or skin from chicken, and eating while watching TV. Usual diet was assessed with a validated diet history. Analyses were performed with linear regression with adjustment for main confounders.

**Results:**

Compared to individuals with ≤1 OREB, those with ≥5 OREB had a higher food energy density (β 0.10; 95% CI 0.08, 0.12 kcal/g/day; p-trend<0.001) and a higher consumption of sugary drinks (β 7; 95% CI −7, 20 ml/day; p-trend<0.05) and of alcoholic beverages (β 24; 95% CI 10, 38 ml/day; p-trend<0.001). Specifically, a higher number of OREB was associated with higher intake of dairy products and red meat, and with lower consumption of fresh fruit, oily fish and white meat. No association was found between the number of OREB and food portion size or the number of eating occasions.

**Conclusions:**

OREB were associated with higher food energy density and higher consumption of sugary and alcoholic beverages. Avoiding OREB may prove difficult because they are firmly socially rooted, but these results may nevertheless serve to palliate the undesirable effects of OREB by reducing the associated energy intake.

## Introduction

The main guidelines for weight control recommend avoiding or moderating the so called obesity-related eating behaviors (OREB), which include skipping breakfast, eating at fast-food restaurants, snacking, and eating while watching television (TV), among others [1]–[4]. In a previous study, we provided support for this recommendation because we showed that individuals with a higher number of OREB had a higher energy intake [5].

Total energy intake can be decomposed into the following constituents [6]: food portion size [7], food energy density (ED) [8]–[10], the number of eating occasions (EO) [11]–[12], and the energy intake from energy-rich beverages, like sugary and alcoholic drinks [13], [14]. To our knowledge, this is the first study to examine the association between the OREB and these energy components. Given that eliminating or moderating OREB may prove difficult because they are firmly socially rooted (e.g., eating at fast-food restaurants or eating while watching TV), this study is important because it may suggest ways to palliate the undesirable effects of OREB by reducing the associated energy intake.

## Subjects and Methods

### Study design and participants

Data were taken from the Study on Nutrition and Cardiovascular Risk in Spain (ENRICA study), whose methods has been reported elsewhere [15]. This is a cross-sectional study conducted from 2008 to 2010 among 12,948 individuals representative of the non-institutionalized Spanish population aged 18 years and older. The study participants were selected by stratified cluster sampling. The sample was first stratified by province (the 50 provinces of Spain) and size of municipality (10,000; 10,000–100,000; 100,000–500,000; >500,000 population). Second, clusters were selected randomly in 2 stages: municipalities and census sections. Finally, the households within each section were selected by random telephone dialing using the directory of telephone land-lines as the sampling frame. Subjects in the households were selected proportionally to the distribution of the population of Spain by sex and age group (18–29, 30–44, 45–64, ≥65 years). Only 1 person was selected in each household; when there was more than one person in the required age and sex group, the invited individual was chosen randomly. Information was obtained from a total of 248 municipalities and 1241 census sections in Spain.

Information was collected in three stages. First, a phone interview on socio-demographic, lifestyle and diagnosed morbidity; second, a home visit to obtain blood and urine samples; and third, another home visit to administer a structured questionnaire on OREB, to obtain a diet history and to measure blood pressure and anthropometric variables.

Study participants provided written informed consent. The ENRICA protocol was approved by the clinical research ethics committees of the University Hospital *La Paz* in Madrid and Hospital *Clinic* in Barcelona.

### Study variables

#### Obesity-related eating behaviors

We used information on 8 self-reported OREB which have been shown to be associated with increased energy intake [5]. We asked participants the following question about planning the amount of food served on the plate: 1) “Before sitting down at the table, do you think about how much you intend to eat?” Other OREB considered were 2) consuming precooked and/or canned foods, 3) buying chocolates or other snacks in vending machines, and 4) eating in fast-food restaurants. Participants also reported whether they had any mindful eating behaviors such as 5) selecting low-energy foods, 6) removing visible fat from meat, and 7) taking the skin off the chicken before eating. To assess meal context, participants were asked 8) how often they had lunch or dinner while watching TV.

#### Diet

We used a computerized diet history, developed from the one used in the EPIC-cohort study in Spain [16], [17], to assess habitual food consumption the previous year. The diet history asked about the food consumed in a typical week, and all foods consumed at least once every 15 days were recorded. Nutrient intake was calculated using standard food composition tables [15]. The diet history collected detailed information on the daily EO, including the three main meals (breakfast, lunch and dinner) and the two intermediate meals (the mid-morning snack or “almuerzo,” and the afternoon snack or “merienda”), which form part of the traditional Spanish diet. Eating between meals (snacking) was considered an additional EO, which in most cases took place after dinner. Thus the range of EO was 0 to 6. Food portion size per EO was obtained by summing the weight (g) of all solid foods consumed divided by the number of EO. Lastly, ED from solid food was calculated as the ratio of the total energy intake (kcal) from solid foods over the total weight of those foods in a week. Beverages were excluded from the calculations because energy intake from beverages is regulated differently from energy intake from solid foods and because ED from solid food has shown stronger associations with weight change than ED from all foods (solid plus liquid) [18].

The diet history also assessed the reported consumption of sugar-sweetened beverages (carbonated and non-carbonated drinks, iced drinks, energy drinks, fruit juices and nectars) and of alcoholic beverages (wine, beer, cider, spirits). Beverage consumption was expressed in ml/day.

#### Other variables

We also considered other variables which could be associated with OREB and with energy intake [19]. Individuals reported sociodemographic variables (sex, age, educational level, occupation-based social class) and lifestyles, including smoking, time spent watching TV and leisure time physical activity, which was assessed with the EPIC-Spain questionnaire and expressed as metabolic equivalent tasks (MET)-hour/week [20].

Weight and height were measured with standardized procedures [21], and we calculated body mass index (BMI) as weight in kg divided by squared height in m.

Information was also used on physician-diagnosed morbidity reported by the participant, including coronary disease, stroke, cancer at any site, and osteomuscular disease (osteoarthritis, rheumatoid arthritis, and hip fracture).

### Statistical analysis

Among the 12,948 participants, we excluded 590 who lacked data on at least one OREB, 206 without diet information, 71 with extreme values on energy intake (<800 to >5000 kcal/day in men; <500 to >4000 kcal/day in women), and 535 without data on other study variables. Thus, the analyses were conducted with 11,546 individuals.

The association between each OREB and portion size, ED and number of EO of solid food, and consumption of sugary and alcoholic beverages was summarized with β coefficients and their 95% confidence intervals, obtained from linear regression. The analyses were also conducted with the number of OREB as the principal independent variable. To ensure a sufficient number of individuals, the OREB were grouped in five categories: ≤1, 2, 3, 4 and ≥5. We tested the linear relationship (P for trend) by modeling the number of OREB as a continuous variable. Regression models were adjusted for sociodemographic and lifestyle variables, BMI, and reported morbidity; moreover, they were adjusted simultaneously for portion size, ED, number of EO, and consumption of sugary and alcoholic drinks, because these variables are usually correlated (table 1).

**Table 1:** Pearson correlation coefficients between food portion size, energy density of food, number of eating occasions, and beverage consumption.

	Energy density of solid food, kcal/g	Eating occasions of solid food, n/day^a^	Sugary beverages, ml/day	Alcoholic beverages, ml/day
**Portion size of solid food, g/EO**	−0.22**	−0.51**	0.01	0.07**
**Energy density of solid food, kcal/g**		0.02	0.22**	0.11**
**Eating occasions of solid food, n/day** a			0.01	−0.08**
**Sugary beverages**				−0.01

** p<0.001;

EO: Eating occasion.

a Eating occasions are breakfast, mid-morning snack, lunch, afternoon snack, dinner, and eating between these meals (in most cases after dinner).

Statistical significance was set at two-sided p<0.05. Statistical analyses were performed with the survey procedures in Stata v.11, StataCorp LP, USA, to account for the complex sampling design [22].

## Results

Among the 11,546 study participants, the portion size (mean ± SD) of solid food was 289±94 g/EO, ED from solid food was 1.5±0.36 kcal/g and the number of EO per day was 4.7±1. Also, they consumed 114±220 ml/day of sugary drinks and 127±241 ml/day of alcoholic beverages.

Table 1 shows the matrix of correlations between the dependent variables in this study. As expected, the food portion size was inversely correlated with food ED and the number of EO. Moreover, food ED showed a direct correlation with the consumption of sugary and alcoholic beverages.

Table 2 shows that the portion size was higher in men and in those with higher physical activity. As regards ED, it was higher in men, younger individuals, and current smokers. Consumption of sugary and alcoholic beverages was higher in men, the youngest population segment, those with university studies, current smokers, and the most physically active. As also shown in table 2, individuals with obesity and reported morbidity generally had a larger portion size, lower ED and lower consumption of sugary drinks. Lastly, while obesity and stroke were associated with higher consumption of alcoholic beverages, coronary disease, asthma, cancer and osteomuscular disease were associated with lower consumption (table 2).

**Table 2:** Portion size, energy density, number of eating occasions, and consumption of sugary and alcoholic beverages, according to the characteristics of the study participants^a^.

	Portion size of solid food g/EO	Energy density of solid food kcal/g	Eating occasions of solid food^b^ n/day	Sugary beverages ml/day	Alcoholic beverages ml/day
**Sex**					
Men	317 (98)	1.60 (0.34)	4.57 (1.01)	138 (241)	194 (288)
Women	262 (81)	1.47 (0.37)	4.82 (1.02)	91 (193)	62 (151)
**Age**, years					
18–44	282 (92)	1.64 (0.35)	4.74 (1.00)	165 (261)	119 (242)
45–64	299 (99)	1.48 (0.35)	4.70 (1.06)	75 (171)	153 (267)
≥65	291 (93)	1.36 (0.30)	4.61 (1.01)	44 (109)	110 (198)
**Educational level**					
Primary school or less	288 (92)	1.48 (0.35)	4.69 (1.01)	89 (190)	118 (235)
Secondary school	289 (94)	1.59 (0.36)	4.69 (1.03)	147 (256)	128 (251)
University	291 (98)	1.52 (0.35)	4.72 (1.03)	92 (186)	137 (234)
**Smoking**					
Never smokers	286 (92)	1.49 (0.35)	4.74 (1.02)	108 (208)	83 (167)
Past smokers	303 (98)	1.49 (0.35)	4.66 (1.03)	88 (187)	170 (270)
Current smokers	282 (93)	1.65 (0.35)	4.66 (1.03)	149 (261)	167 (302)
**Social class**					
Manual workers	291 (97)	1.52 (0.36)	4.66 (1.03)	104 (213)	127 (236)
Non-manual workers	287 (92)	1.56 (0.36)	4.75 (1.01)	127 (230)	128 (249)
**Physical activity**, METs-h/week					
Tertile 1 (<16.5)	283 (93)	1.56 (0.37)	4.67 (1.05)	109 (216)	122 (261)
Tertile 2 (≥16.5 to <33)	288 (95)	1.50 (0.36)	4.71 (1.03)	98 (118)	120 (218)
Tertile 3 (≥33)	295 (95)	1.55 (0.34)	4.72 (0.99)	134 (226)	139 (243)
**Time spent watching TV**, h/week					
Tertile 1 (<7)	292 (97)	1.55 (0.36)	4.73 (1.05)	118 (211)	123 (236)
Tertile 2 (≥7 to <14)	289 (91)	1.54 (0.36)	4.67 (1.02)	122 (242)	131 (244)
Tertile 3 (≥14)	288 (95)	1.52 (0.36)	4.70 (1.01)	108 (212)	127 (242)
**Body mass index**, kg/m^2^					
<25	280 (92)	1.57 (0.36)	4.73 (1.01)	132 (240)	95 (194)
25–29.9	295 (97)	1.52 (0.35)	4.67 (1.04)	102 (195)	147 (257)
≥30	296 (93)	1.51 (0.37)	4.68 (1.01)	107 (227)	147 (275)
**Coronary heart disease**					
No	289 (94)	1.54 (0.36)	4.70 (1.02)	114 (221)	128 (242)
Yes	315 (107)	1.36 (0.32)	4.49 (0.97)	35 (65)	76 (169)
**Stroke**					
No	289 (94)	1.59 (0.36)	4.70 (1.02)	115 (221)	127 (241)
Yes	301 (98)	1.43 (0.31)	4.49 (0.98)	59 (97)	149 (271)
**Asthma**					
No	289 (94)	1.54 (0.36)	4.70 (1.03)	114 (221)	129 (245)
Yes	282 (96)	1.54 (0.35)	4.73 (0.97)	112 (209)	99 (190)
**Cancer at any site**					
No	289 (94)	1.54 (0.36)	4.70 (1.02)	115 (221)	128 (241)
Yes	293 (105)	1.44 (0.33)	4.73 (0.94)	61 (129)	97 (241)
**Osteomuscular disease**					
No	290 (95)	1.57 (0.36)	4.69 (1.03)	127 (232)	133 (248)
Yes	286 (93)	1.41 (0.34)	4.73 (1.01)	66 (159)	105 (213)

EO: Eating occasion; SD: Standard deviation

a Values are means (standard deviation).

b Eating occasions are breakfast, mid-morning snack, lunch, afternoon snacks, dinner and eating between these meals (in most cases after dinner).

Eating precooked food and rarely removing visible fat from meat or skin from chicken were associated with higher portion size, while not planning the amount of food to eat was inversely associated (table 3). All OREB were associated with a slightly higher ED, with the exception of not planning the amount of food to eat, which showed an inverse association. Individuals who frequently ate precooked foods, bought snacks at vending machines and ate while watching TV showed a higher number of EO, but those who frequently ate at fast-food restaurants and who rarely chose low-energy foods had a lower number of EO (table 3).

**Table 3:** Association of individual obesity-related eating behaviors with portion size, energy density of foods, number of eating occasion, and beverage consumption^a^.

	Portion size of solid food, g/EO	Energy density of solid food, kcal/g	Eating occasions of solid food, n/day	Sugary beverages, ml/day	Alcoholic beverages, ml/day
**Planning how much to eat before sitting down**					
Yes	Ref.	Ref.	Ref.	Ref.	Ref.
No	−8 (−12, −5)**	−0.03 (−0.04, −0.01)*	0.02 (−0.03, 0.07)	16 (6, 25)*	3 (−8, 14)
**Eating precooked/canned food**					
<1 time/wk	Ref.	Ref.	Ref.	Ref.	Ref.
≥1 time/wk	6 (3, 9)*	0.05 (0.03, 0.06)**	0.27 (0.23, 0.30)**	5 (−5, 14)	10 (−9, 20)
**Buying snacks at vending machines**					
<1 time/wk	Ref.	Ref.	Ref.	Ref.	Ref.
≥1 time/wk	−3 (−11, 4)	0.10 (0.07, 0.14)**	0.18 (0.09, 0.26)**	10 (−14, 35)	−10 (−39, 18)
**Eating at fast-food restaurants**					
<1 time/wk	Ref.	Ref.	Ref.	Ref.	Ref.
≥1 time/wk	−3 (−9, 3)	0.11 (0.08, 0.13)**	−0.09 (−0.15, −0.02)*	69 (45, 91)**	−21 (−46, 3)
**Choosing low-energy foods**					
Frequently/always/sometimes	Ref.	Ref.	Ref.	Ref.	Ref.
Never/almost never	3 (−0.35, 7)	0.10 (0.09, 0.12)**	−0.10 (−0.14, −0.06)**	6 (−5, 16)	20 (9, 31)**
**Removing visible fat from meat** b					
Frequently/always/sometimes	Ref.	Ref.	Ref.	Ref.	Ref.
Never/almost never	7 (1, 13)*	0.06 (0.04, 0.08)**	−0.03 (−0.09, 0.02)	12 (−4, 28)	11 (−6, 28)
**Removing skin from chicken** c					
Frequently/always/sometimes	Ref.	Ref.	Ref.	Ref.	Ref.
Never/almost never	5 (0.22, 10)*	0.06 (0.04, 0.08)**	0.02 (−0.03, 0.07)	6 (−10, 24)	15 (−2, 32)
**Eating while watching TV**					
≤2 times/wk	Ref.	Ref.	Ref.	Ref.	Ref.
>2 times/wk	2 (−2, 6)	0.04 (0.02, 0.05)**	0.05 (0.01, 0.09)*	19 (10, 29)**	4 (−8, 15)

N = 11,546.

* p<0.05;

** p<0.001;

EO: Eating occasion.

a Values are β (95% CI) obtained from linear regression and adjusted for sex, age, educational level, smoking, social class, leisure time physical activity, time spent watching TV, body mass index (<25, 25–29.9, ≥30 kg/m^2^), coronary disease, stroke, asthma, cancer, osteomuscular disease, portion size of solid food, energy density of solid food, number of EO of solid food, consumption of sugary beverages, and consumption of alcoholic beverages, when appropriate.

b Analyses based on 10,154 participants who eat meat.

c Analyses based on 9,808 participants who eat chicken.

As regards consumption of beverages, not planning the amount of food to eat, eating at fast-food restaurants and eating while watching TV were associated with higher intake of sugary drinks. Moreover, those who rarely chose low energy food had a higher consumption of alcoholic beverages (table 3).

An increasing number of OREB was associated with increasing ED from solid food, and with increasing consumption of sugary and alcoholic beverages (table 4). Compared to individuals with ≤1 OREB, those with ≥5 OREB had a higher ED (β 0.10; 95% CI 0.08, 0.12 kcal/g/day; p-trend<0.001) and a higher intake of sugary drinks (β 7; 95% CI −7, 20 ml/day; p-trend<0.05) and of alcoholic beverages (β 24; 95% CI 10, 38 ml/day; p-trend<0.001). No clear association was found between the number of OREB and food portion size or the number of EO.

**Table 4:** Association of the number of obesity-related eating behaviors with portion size, energy density of foods, number of eating occasions, and beverage consumption.

	Number of obesity-related eating behaviors^a^					
	≤1 (n = 1,621)	2 (n = 2,314)	3 (n = 2,439)	4 (n = 1,578)	≥5 (n = 3,594)	P-trend
**Portion size of solid food**, *g/EO, mean (SD)*	289 (94)	292 (96)	291 (93)	293 (95)	285 (94)	
β (95% CI)^b^	Ref.	2 (−4, 7)	3 (−2, 8)	1 (−5, 6)	4 (−1, 10)	0.138
**Energy density of solid food**, *kcal/g, mean (SD)*	1.43 (0.34)	1.47 (0.35)	1.55 (0.34)	1.58 (0.36)	1.60 (0.36)	
β (95% CI)^b^	Ref.	0.01 (−0.01, 0.03)	0.07 (0.05, 0.09)**	0.08 (0.05, 0.10)**	0.10 (0.08, 0.12)**	<0.001
**Eating occasions of solid food**, *n/day, mean (SD)*	4.73 (1.06)	4.66 (1.01)	4.65 (1.00)	4.60 (1.02)	4.78 (1.02)	
β (95% CI)^b^	Ref.	−0.04 (−0.10, 0.02)	−0.03 (−0.10, 0.03)	−0.09 (−0.16, −0.02)*	0.07 (0.01, 0.13)*	0.008
**Sugary beverages**, *ml/day, mean (SD)*	89 (200)	97 (201)	107 (224)	138 (255)	131 (219)	
β (95% CI)^b^	Ref.	−4 (−18, 9)	−7 (−22, 7)	15 (−3, 33)	7 (−7, 20)	<0.05
**Alcoholic beverages**, *ml/day, mean (SD)*	102 (193)	110 (22)	132 (250)	136 (258)	143 (253)	
β (95% CI)^b^	Ref.	4 (−10, 17)	16 (2, 30)*	9 (−9, 26)	24 (10, 38)*	<0.001

N = 11,546.

* p<0.05;

** p<0.001;

EO: Eating occasion; SD: Standard deviation.

a Obesity-related eating behaviors are as follows: not planning how much to eat before sitting down, consuming precooked and/or canned foods ≥1 time/wk, buying snacks at vending machines ≥1 time/wk, eating at fast-food restaurants ≥1 time/wk, never or almost never choosing low-energy foods, never or almost never removing visible fat from meat, never or almost never removing skin from chicken, and eating while watching TV >2 times/wk.

b Adjusted for sex, age, educational level, smoking, social class, leisure time physical activity, time spent watching TV, body mass index (<25, 25–29.9, ≥30 kg/m^2^), coronary disease, stroke, asthma, cancer, osteomuscular disease, portion size of solid food, energy density of solid food, number of EO of solid food, consumption of sugary beverages, and consumption of alcoholic beverages, when appropriate.

To further understand the association between the number of OREB and ED, we calculated the Pearson correlation coefficients between ED and consumption of individual food groups. The types of food most positively associated with ED were sweets, bread, sausages, cheese and other dairy products, pasta and potatoes; the foods most inversely correlated were fresh fruits, vegetables, white fish, legumes, oily fish and white meat (table 5). The number of OREB was then regressed on the consumption of those foods most strongly associated (either positively or negatively) with ED. An increasing number of OREB was associated with an increasing consumption of cheese and other dairy products and red meat (table 6), and with a decreasing consumption of fresh fruits, oily fish and white meat (p-trend<0.05 in all cases but red meat) (table 7). We also examined the association between the number of OREB and consumption of the main types of sugary and alcoholic beverages. We found a positive dose-response relationship (p-trend<0.001) between the number of OREB and the consumption of sugar-sweetened soft drinks and beer (table 8).

**Table 5:** Top positive and negative Pearson correlations coefficients between food groups and total energy density from solid food^a^.

Positive			Negative		
Rank	Food group	Pearson correlation coefficient	Rank	Food group	Pearson correlation coefficient
**1**	Sweets^b^	0.48	**1**	Fresh fruits^i^	−0.56
**2**	Bread^c^	0.30	**2**	Vegetables^j^	−0.45
**3**	Sausages^d^	0.29	**3**	White fish^k^	−0.14
**4**	Cheese and other dairy products^e^	0.16	**4**	Legumes^l^	−0.10
**5**	Pasta^f^	0.16	**5**	Oily fish^m^	−0.08
**6**	Potatoes^g^	0.13	**6**	White meat^n^	−006
**7**	Red meat^h^	0.09			

N = 11,546.

a Results are shown only for food groups with Pearson correlation coefficient >0.05.

b Jam, chocolate pudding, chocolate truffles, chocolate-hazelnut creams, nougats, marzipan, cakes, sponge cakes, croissants, donuts, pastries and cookies.

c White bread, wholemeal bread, breadsticks, hamburger and hotdog buns.

d Pork sausages, veal sausages, and poultry sausages.

e Unripened cheese, ripened cheese, processed cheese, yogurt, custard, mousse, and ice cream.

f Unstuffed pasta, stuffed pasta, and pizza.

g Baked potatoes, boiled potatoes, mashed potatoes, French fries, and potato chips.

h Veal, beef, pork, wild boar, horse, lamb and goat..

i Berries, custard apple, apple, pear, plum, pomegranate, passion fruit, fig, kiwi, lychee, lime, lemon, tangerine, orange, mango, peach, nectarine, apricot, loquat, persimmon, watermelon, papaya, and pineapple.

j Chard, celery, watercress, collard green, borage, spinach, cabbage, endive, lettuce, thistle, scallion, fennel, onion, leek, garlic, asparagus, palm heart, turnip, parsnip, radishes, beets, soy, carrot, artichoke, eggplant, broccoli, cauliflower, zucchini, pumpkin, green been, corn, pepper, tomato, champignon, and mushroom.

k Pollack, weever, blue whiting, cod, sea bream, red scorpionfish, dogfish, black seabream, pouting, megrim, halibut, common sole, seabass, whiting, hake, grouper, flathead mullet, common pandora, young hake, catshark, plaice, angler, blonde ray, turbot, red mullet, and white seabream.

l Chickpeas, beans, and lentils.

m Anchovy, sardine, eel, herring, tuna, albacore, Atlantic horse mackerel, Atlantic mackerel, transparent goby, conger, swordfish, pomfret, and salmon.

n Chicken, quail, pheasant, goose, duck, turkey, pigeon, partridge and rabbit.

**Table 6:** Association of the number of obesity-related eating behaviors with individual foods groups associated with higher energy density (ED).

	Number of obesity-related eating behaviors^a^					
	≤1 (n = 1,621)	2 (n = 2,314)	3 (n = 2,439)	4 (n = 1,578)	≥5 (n = 3,594)	P-trend
**Foods groups associated with higher ED**						
**Sweets**, *g/day, mean (SD)*	52 (60)	54 (62)	58 (60)	60 (69)	65 (62)	
β (95% CI)^b^	Ref.	0.54 (−3.13, 4.21)	−2.51 (−6.39, 1.36)	−1.30 (−5.70, 3.11)	0.34 (−3.28, 3.96)	0.922
**Bread**, *g/day, mean (SD)*	148 (90)	153 (88)	160 (90)	157 (92)	166 (95)	
β (95% CI)^b^	Ref.	2.35 (−3.24, 7.94)	1.71 (−4.20, 7.61)	−4.06 (−10.72, 2.59)	2.79 (−3.09, 8.69)	0.771
**Sausages**, *g/day, mean (SD)*	43 (54)	45 (52)	50 (52)	55 (60)	50 (53)	
β (95% CI)^b^	Ref.	−0.32 (−3.80, 3.73)	0.91 (−3.09, 4.91)	3.29 (−1.39, 7.97)	−2.38 (−5.86, 1.09)	0.211
**Cheese and other dairy**, *g/day, mean (SD)*	65 (79)	69 (75)	75 (80)	77 (82)	85 (80)	
β (95% CI)^b^	Ref.	4.26 (−0.93, 9.44)	6.12 (1.01, 11.21)*	6.77 (0.60, 12.93)*	12.7 (7.62, 17.85)**	<0.001
**Pasta**, *g/day, mean (SD)*	38 (35)	42 (39)	46 (39)	50 (46)	48 (40)	
β (95% CI)^b^	Ref.	1.91 (−0.48, 4.31)	2.91 (0.57, 5.27)*	5.18 (1.89, 8.46)*	2.22 (−0.12, 4.57)	0.092
**Potatoes**, *g/day, mean (SD)*	48 (43)	48 (40)	49 (41)	51 (42)	48 (36)	
β (95% CI)^b^	Ref.	−1.22 (−3.95, 1.51)	−1.80 (−4.74, 1.13)	−0.36 (−3.62, 2.91)	−4.65 (−7.33, −1.97)*	<0.001
**Red meat**, *g/day, mean (SD)*	29 (33)	32 (34)	34 (37)	37 (40)	34 (35)	
β (95% CI)^b^	Ref.	2.70 (0.61, 4.78)*	4.05 (1.80, 6.30)**	4.83 (2.11, 7.55)**	2.36 (0.25, 4.48)*	0.130

N = 11,546.

* p<0.05;

** p<0.001;

EO: Eating occasion; SD: Standard deviation.

a Obesity-related eating behaviors are as follows: not planning how much to eat before sitting down, consuming precooked and/or canned foods ≥1 time/wk, buying snacks at vending machines ≥1 time/wk, eating at fast-food restaurants ≥1 time/wk, never or almost never choosing low-energy foods, never or almost never removing visible fat from meat, never or almost never removing skin from chicken, and eating while watching TV >2 times/wk.

b Adjusted for sex, age, educational level, smoking, social class, leisure time physical activity, time spent watching TV, body mass index (<25, 25–29.9, ≥30 kg/m^2^), coronary disease, stroke, asthma, cancer, osteomuscular disease, portion size of solid food, energy density of solid food, number of EO of solid food, consumption of sugary beverages, and consumption of alcoholic beverages, when appropriate.

**Table 7:** Association of the number of obesity-related eating behaviors with individual foods groups associated with lower energy density (ED).

	Number of obesity-related eating behaviors^a^					
	≤1 (n = 1,621)	2 (n = 2,314)	3 (n = 2,439)	4 (n = 1,578)	≥5 (n = 3,594)	P-trend
**Foods groups associated with lower ED**						
**Fresh fruits**, *g/day, mean (SD)*	278 (198)	257 (185)	225 (175)	204 (173)	214 (169)	
β (95% CI)^b^	Ref.	−0.75 (−11.30, 9.81)	−7.75 (−18.03, 2.53)	−16.68 (−28.51, −4.82)*	−8.76 (−18.78, 1.26)	0.014
**Vegetables**, *g/day, mean (SD)*	218 (140)	210 (148)	198 (129)	196 (133)	190 (122)	
β (95% CI)^b^	Ref.	−1.58 (−10.3, 7.21)	0.53 (−7.84, 8.90)	0.41 (−8.92, 9.74)	−3.68 (−11.55, 4.19)	0.389
**White fish**, *g/day, mean (SD)*	28 (36)	27 (32)	25 (43)	24 (30)	25 (28)	
β (95% CI)^b^	Ref.	−0.64 (−3.02, 1.75)	−1.17 (−3.75, 1.41)	−1.28 (−3.83, 1.27)	−0.96 (−3.28, 1.35)	0.433
**Legumes**, *g/day, mean (SD)*	57 (71)	54 (63)	54 (61)	52 (57)	54 (58)	
β (95% CI)^b^	Ref.	−2.13 (−6.68, 2.41)	−0.55 (−5.48, 4.38)	−2.60 (−7.67, 2.47)	−1.39 (−5.76, 2.99)	0.680
**Oily fish**, *g/day, mean (SD)*	19 (27)	19 (26)	17 (23)	18 (23)	17 (20)	
β (95% CI)^b^	Ref.	0.08 (−1.58, 1.74)	−1.17 (−2.80, 0.46)	−0.54 (−2.39, 1.30)	−2.00 (−3.55, −0.46)*	0.002
**White meat**, *g/day, mean (SD)*	39 (36)	42 (41)	42 (40)	42 (37)	35 (35)	
β (95% CI)^b^	Ref.	2.65 (0.05, 5.24)*	2.47 (−0.19, 5.13)	1.64 (−1.11, 4.40)	−4.03 (−6.54, −1.52)*	<0.001

N = 11,546.

* p<0.05;

** p<0.001;

EO: Eating occasion; SD: Standard deviation.

a Obesity-related eating behaviors are as follows: not planning how much to eat before sitting down, consuming precooked and/or canned foods ≥1 time/wk, buying snacks at vending machines ≥1 time/wk, eating at fast-food restaurants ≥1 time/wk, never or almost never choosing low-energy foods, never or almost never removing visible fat from meat, never or almost never removing skin from chicken, and eating while watching TV >2 times/wk.

b Adjusted for sex, age, educational level, smoking, social class, leisure time physical activity, time spent watching TV, body mass index (<25, 25–29.9, ≥30 kg/m^2^), coronary disease, stroke, asthma, cancer, osteomuscular disease, portion size of solid food, energy density of solid food, number of EO of solid food, consumption of sugary beverages, and consumption of alcoholic beverages, when appropriate.

**Table 8:** Association of the number of obesity-related eating behaviors with the main type of sugary and alcoholic beverages.

	Number of obesity-related eating behaviors^a^					
	≤1 (n = 1,621)	2 (n = 2,314)	3 (n = 2,439)	4 (n = 1,578)	≥5 (n = 3,594)	P-trend
**Sugary beverages**						
**Sugar-sweetened soft drinks**, *ml/day, mean(SD)*	56 (161)	63 (166)	76 (194)	97 (217)	94 (194)	
β (95% CI)^b^	Ref.	−2 (−14, 9)	−2 (−14, 11)	13 (−3, 28)	11 (−1, 22)	<0.001
**Juices and nectars**, *ml/day, mean (SD)*	33 (114)	33 (99)	32 (99)	42 (118)	36 (96)	
β (95% CI)^b^	Ref.	2 (−9, 6)	−6 (−14, 2)	2 (−7, 11)	−4 (−11, 3)	0.482
**Alcoholic beverages**						
**Beer**, *ml/day, mean (SD)*	41 (128)	52 (162)	77 (205)	75 (205)	90 (217)	
β (95% CI)^b^	Ref.	6 (−4, 16)	22 (11, 34)**	12 (−2, 25)	31 (20, 42)**	<0.001
**Wine**, *ml/day, mean (SD)*	59 (133)	53 (139)	50 (126)	54 (139)	48 (112)	
β (95% CI)^b^	Ref.	−4 (−12, 5)	−7 (−16, 1)	−5 (−16, 5)	−8 (−16, −1)	0.053
**Spirits**, *ml/day, mean (SD)*	2 (13)	4 (15)	4 (17)	7 (25)	5 (18)	
β (95% CI)^b^	Ref.	1 (−0.14, 2)	1 (−1, 2)	2 (1, 4)*	1 (−0.01, 2)	0.043

N = 11,546.

* p<0.05;

** p<0.001;

EO: Eating occasion; SD: Standard deviation.

a Obesity-related eating behaviors are as follows: not planning how much to eat before sitting down, consuming precooked and/or canned foods ≥1 time/wk, buying snacks at vending machines ≥1 time/wk, eating at fast-food restaurants ≥1 time/wk, never or almost never choosing low-energy foods, never or almost never removing visible fat from meat, never or almost never removing skin from chicken, and eating while watching TV >2 times/wk.

b Adjusted for sex, age, educational level, smoking, social class, leisure time physical activity, time spent watching TV, body mass index (<25, 25–29.9, ≥30 kg/m^2^), coronary disease, stroke, asthma, cancer, osteomuscular disease, portion size of solid food, energy density of solid food, number of EO of solid food, consumption of sugary beverages, and consumption of alcoholic beverages, when appropriate.

Lastly, those individuals with ≥5 OREB compared those with ≤1 OREB had a higher BMI (β 0.45; 95% CI 0.12, 0.78 kg/m^2^; p-trend<0.05). Also, those with ≥5 OREB ingested 248 kcal/day more than those with ≤1 OREB. Of this excess energy intake, 85.8% came from solid food, 5.2% from sugary drinks and 9.0% from alcoholic beverages.

## Discussion

Our results show that a higher number of OREB is associated with higher ED from solid food and higher consumption of sugary and alcoholic beverages. Specifically, OREB were associated with higher intake of cheese and other dairy, red meat, sugar-sweetened soft drinks and beer, and with lower consumption of fresh fruit, oily fish and white meat.

The ED from solid food in Spain was lower than in the US (2.05 kcal/g in 2003–2006) [6], probably because the Spanish diet continues to include a large amount of vegetables and fruit, which is characteristic of the Mediterranean dietary pattern [23]. As expected, portion size per EO was inversely associated with ED, so that portion size in Spain was higher than in the US [6]. Moreover, less than 1% of the Spanish population skips breakfast, and midmorning or afternoon snacks are quite frequent [19], so that the number of EO (meals plus eating between meals) in Spain was similar to that in the US, where it was 4.9 EO/day in 2003–2006 [6]. In fact, the larger number of EO seems to be the factor that has contributed most to the increase in energy intake underlying the obesity epidemic in the US from 1976 to 2006 [6]. Lastly, consumption of sugary drinks in Spain was similar to that in some Anglo-Saxon countries like the United Kingdom [24], but still lower than in the US [25], [26].

Some of the associations between OREB and the components of energy intake are quite intuitive For instance, frequently buying chocolates and other snacks in vending machines and eating at fast-food restaurants, and rarely choosing low-energy foods showed the strongest associations with higher ED from solid food. Also, as expected, eating at fast-food restaurants and eating while watching TV had the strongest association with higher consumption of sugary drinks. These observations provide additional biological plausibility to the relationship between OREB and excess weight because there is substantial evidence that increasing ED from solid food [18] and intake of sugary drinks [27], [28] is associated with weight gain.

Previous research has also found an association between several OREB, ED and consumption of sugary drinks. Specifically, in Spanish adults, frequent fast-food consumption has been linked to increased ED and energy intake [29]. Also, in US children and adults, the consumption of sugary drinks has been found to be higher in those who used vending machines and consumed fast-food [30], [31], and in those who frequently ate while watching TV [32], [33].

Although we found no clear association between the number of OREB and EO, a few OREB did show such an association. Specifically, eating precooked food, buying snacks in vending machines and eating while watching TV were associated with a higher number of EO. The relevance of these associations is uncertain, because eating frequency has not been consistently associated with obesity [35]. Nevertheless some of our results are in line with previous research, because eating while watching TV has been related to snacking, which may increase the number of EO [36].

Not choosing low-energy foods was the only OREB to be statistically associated with higher consumption of alcoholic drinks, but most of the other OREB also showed a tendency towards higher consumption. As a result, the number of OREB showed a dose-response relation with the consumption of alcoholic beverages. In previous research, alcohol intake has been linked with fast-food consumption [31] and with eating while watching TV [33], [34]. Of note is that, although alcohol intake has been linked to increased energy intake [37], and drinking alcohol with meals has been linked to poor adherence to dietary guidelines [38], the association between alcohol intake and obesity is still uncertain [39].

Given that the magnitude of the association between OREB and energy intake was small, the association between OREB and higher food ED and consumption of sugary and alcoholic drinks should necessarily be small. However, even small associations may suffice to alter energy balance and produce obesity in the long term [40]. In the Nurses' Health Study, an increase of 0.25 kcal/g in ED from solid food (about double the ED associated with ≥5 OREB in our study) was associated with a 5-kg gain over 8 years of follow-up [18]. As for sugary drinks, an increase of a 12-ounce serving (about 9 times the amount of sugary drinks associated with ≥5 OREB) was linked to a gain of 0.6 kg over 4 years in three separate US cohorts [27].

This work has several strengths and limitations. Among the strengths is the large study sample, which is representative of the population of an entire country. Also, diet was measured with a validated diet history. Lastly, the analyses controlled for a good number of confounders. The most important limitation is the cross-sectional design, which does not permit causal inferences for the observed associations. Another limitation was that, as in previous research in this field [35], we lacked standardized definitions of OREB and rigorously validated questionnaires to assess them; moreover OREB were self-reported, which may lead to underestimation of the true frequency of the OREB, because of recall or desirability bias. The most likely effect on the study results is underestimation of the true association between OREB and energy intake and its drivers (e.g., portion size, sugary drinks, etc.).

Lastly, our results are of practical importance because they support healthy-diet guidelines recommending to moderate OREB. Also, our results suggest possible ways to reduce excess energy intake associated with OREB. The first one is to augment consumption of low-ED food associated with lower weight gain, like fresh fruit or vegetables or soups; at the same time, to reduce high-ED food, like processed meat, and to substitute high-fat for low-fat dairy [10]. The second one is to replace sugary drinks with non-caloric beverages, in particular water, and to a lesser extent with non-sweetened coffee or tea, low-fat milk, and artificially-sweetened beverages [41]. And the third way is to reduce alcohol intake, particularly in the form of beer because it is the most frequently consumed alcoholic beverage and the one most strongly associated with excess energy intake in our study. These changes in food and beverage consumption would reduce energy intake while maintaining or even improving overall diet quality.
